# Overcoming Treatment Challenges in HIV-Associated Mycobacterial Diseases: New Therapeutic Frontiers

**DOI:** 10.3390/ijms262110325

**Published:** 2025-10-23

**Authors:** Omid Nikjeh, Seyedehparmis Rejali, Kayvan Sasaninia, Vishwanath Venketaraman

**Affiliations:** College of Osteopathic Medicine of the Pacific, Western University of Health Sciences, Pomona, CA 91766, USA; omid.nikjeh@westernu.edu (O.N.); seyedehparmis.rejali@westernu.edu (S.R.); kayvan.sasaninia@westernu.edu (K.S.)

**Keywords:** HIV, tuberculosis, *Mycobacterium avium* complex, leprosy, antiretroviral therapy, host-directed therapies, drug resistance, immune reconstitution inflammatory syndrome, host-directed and adjunctive therapeutics, adherence

## Abstract

For drug-susceptible TB, the WHO-endorsed first-line regimen (isoniazid, rifampicin, ethambutol, pyrazinamide) remains the global reference. Therapy must always be tailored to drug susceptibility, especially in MDR- and XDR-TB. HIV-associated mycobacterial infections—including *Mycobacterium tuberculosis* (TB), disseminated *Mycobacterium avium* complex (MAC), and Mycobacterium *leprae* (*M. leprae*)—remain leading causes of morbidity and mortality in people living with HIV (PLWH). TB continues to account for the highest burden of AIDS-related deaths worldwide, while MAC and leprosy complicate care in advanced immunosuppression. This review synthesizes current evidence on epidemiology, clinical features, and management challenges of HIV–mycobacterial co-infections. We discuss drug-susceptible and drug-resistant TB therapies, drug–drug interactions with antiretroviral therapy (ART), and the clinical impact of immune reconstitution inflammatory syndrome (IRIS). Beyond established regimens, we highlight host-directed strategies such as metformin, glutathione augmentation, mTOR modulation, and vitamin D; immunotherapies including interferon-γ, GM-CSF, and IL-7; and therapeutic vaccines (M72/AS01E, MTBVAC, VPM1002) as promising adjuncts. Distinct from guideline-focused overviews, this review emphasizes non-tuberculous mycobacterial disease (NTM, including MAC) and leprosy in PLWH and synthesizes host-directed and adjunctive strategies with their translational prospects, including ART compatibility and IRIS. By integrating TB, NTM, and leprosy across the HIV care continuum, we highlight opportunities not treated in detail elsewhere—particularly HDT-enabled approaches and implementation considerations in PLWH.

## 1. Introduction

HIV co-infection with mycobacterial diseases remains a major global health challenge. Tuberculosis (TB) is the leading cause of death in people living with HIV (PLWH), accounting for a substantial proportion of AIDS-related mortality worldwide [1,2]. Compared with HIV-negative individuals, PLWH have a markedly higher risk of progressing to active TB and experience poorer outcomes, including increased mortality during TB treatment [1].

Disseminated *Mycobacterium avium* complex (MAC) disease, historically frequent in advanced AIDS, declined in incidence with combination antiretroviral therapy (ART) and targeted prophylaxis but continues to cause morbidity in patients with CD4 <50/µL or unsuppressed viremia (Table 1) [1]. Leprosy (*Mycobacterium leprae*) is comparatively uncommon in PLWH and overall susceptibility is not clearly increased; however, ART initiation can unmask subclinical disease or precipitate inflammatory reactions as part of immune reconstitution inflammatory syndrome (IRIS), necessitating timely recognition and management [1].

The management of HIV-associated mycobacterial infections is complicated by prolonged multidrug regimens, overlapping toxicities, drug–drug interactions between antimycobacterial agents and ART, and the risk of IRIS after ART initiation; the added burden of multidrug-resistant (MDR) and extensively drug-resistant (XDR) TB further intensifies these challenges [6]. For drug-susceptible TB, global practice relies on a rifamycin-based regimen with isoniazid, pyrazinamide, and ethambutol over six months, coordinated with ART to mitigate pharmacokinetic interactions and monitor for TB-IRIS [1,6]. For drug-resistant TB, individualized second-line regimens commonly incorporate agents such as bedaquiline and linezolid—often with a fluoroquinolone—according to programmatic and guideline-based frameworks [1]. For disseminated MAC, recommended therapy combines a macrolide with ethambutol, with or without rifabutin, for ≥12 months and until durable immune recovery; primary prophylaxis is reserved for patients with CD4 <50/µL who are not virologically suppressed [6,7]. For leprosy, World Health Organization multidrug therapy (rifampicin, dapsone, clofazimine) remains effective and generally requires no modification in the setting of HIV co-infection [1,6,7].

This review emphasizes that bedaquiline (introduced 2012), delamanid (2015), and pretomanid (2019) are now established components of MDR-TB regimens rather than “novel” drugs in 2025, whereas fluoroquinolones have been integral to TB care since the 1990s [3]. In parallel, host-directed and immunomodulatory approaches—including glutathione augmentation, metformin, mTOR pathway modulation, interferon-γ, GM-CSF, and IL-7—are under investigation as adjuncts to antimicrobial therapy and immune recovery [1,3]. Considering their distinct interactions with ART and IRIS in advanced immunosuppression, inclusion of MAC and *M. leprae* alongside TB remains clinically relevant in the modern ART era [6,7].

## 2. Epidemiology & Clinical Features of Major Mycobacterial Co-Infections in HIV

Mycobacterial infections represent a significant category of opportunistic infections (OIs) linked to HIV. Among these, MAC and *Mycobacterium tuberculosis* are the most prevalent pathogens affecting individuals with HIV. The interaction between HIV and mycobacterial infections complicates disease progression, treatment, and outcomes, necessitating tailored therapeutic approaches.

### 2.1. Tuberculosis (TB) in HIV

#### 2.1.1. Epidemiology of TB in HIV

*M. tuberculosis* typically affects individuals in the earlier stages of HIV infection, responds well to standard treatments, and is the most transmissible among the life-threatening pathogens associated with HIV. The WHO estimates that individuals with HIV account for approximately 8–13% of global tuberculosis cases, with the highest prevalence in sub-Saharan Africa and Southeast Asia. People living with HIV (PLWH) have an estimated 18 (15–21) times higher risk of developing active tuberculosis compared to those without HIV and face a 5–10% annual risk of latent TB reactivation [1,6].

#### 2.1.2. Clinical Features of TB in HIV

TB patients with HIV present systemic immune activation, increased HIV viral load, more severe clinical presentations and reduced success of TB therapy [7]. The clinical presentation of tuberculosis in people living with HIV (PLWH) depends on their CD4 T-cell count. When CD4 levels are above 350/μL, lung lesions resemble those seen in HIV-negative individuals, often featuring upper lobe infiltrates and cavity formation. However, with lower CD4 counts, particularly below 50/μL, extrapulmonary TB becomes more prevalent, sometimes occurring alongside pulmonary TB, and accounts for 40–80% of cases. While the clinical presentation of pulmonary tuberculosis differs in individuals with HIV-1 compared to those without, the most common symptoms still include cough, fever, night sweats, and significant weight loss. Compared to HIV-negative patients, weight loss and fever are more prevalent in HIV-positive individuals, while hemoptysis is less common [3]. In a study conducted in Brazil, weight loss was reported more frequently in patients with a CD4 count below 200 cells/µL compared to those with higher CD4 counts. This symptom was found to help distinguish pulmonary tuberculosis from other pulmonary infections, such as *Pneumocystis jirovecii* pneumonia and bacterial pneumonia, in HIV-1-infected patients [2]. Translational handoff: The next section focuses on host-directed and adjunctive strategies—chosen for ART compatibility, IRIS risk, and feasibility in PLWH—rather than reiterating guideline regimens.

### 2.2. Mycobacterium avium Complex (MAC) in HIV

#### 2.2.1. Epidemiology of MAC in HIV

Disseminated MAC disease usually affects individuals with HIV who have CD4 counts below 50 cells/mm^3^. The incidence of disseminated MAC disease in these individuals, in the absence of effective ART or chemoprophylaxis, has previously been reported to range from 20% to 40% in those with advanced immunosuppression [4].

#### 2.2.2. Clinical Features of MAC in HIV

In individuals with HIV and advanced immunosuppression who are not receiving ART, MAC disease typically presents as a disseminated infection affecting multiple organs, although localized disease can also occur. Early symptoms may be mild and can appear weeks before the onset of mycobacteremia or positive tissue cultures [4].

The clinical presentation of disseminated MAC disease is non-specific. Common findings include fever (over 80%), night sweats (over 35%), weight loss (over 25%), abdominal pain, diarrhea, mesenteric lymphadenopathy, anemia, elevated alkaline phosphatase, and increased lactate dehydrogenase levels [5]. 

While MAC infection is common in immunocompromised individuals, the typical presentation is extrapulmonary. Pulmonary MAC infection is very rare, and most cases occur in patients with pre-existing structural lung disease. Over the past 17 years, there have been no reported cases of isolated pulmonary MAC in HIV/AIDS patients without any underlying structural lung disease [8]. Adjunctive considerations in MAC. Because durable control of disseminated MAC in PLWH depends on immune reconstitution with ART, adjunctive approaches that support myeloid function and limit inflammatory toxicity may improve clinical trajectories, especially when rifamycin use is constrained by DDIs. Practical adjuncts and their translational fit for PLWH are outlined in Section 7.

### 2.3. Leprosy (M. leprae) in HIV

#### 2.3.1. Epidemiology of *M. leprae* in HIV

Leprosy is a chronic, contagious infectious disease caused by *Mycobacterium leprae*. It is found worldwide, particularly in tropical and subtropical regions. Many countries have reported frequent co-occurrence of leprosy and other diseases, including HIV. During the early years of the HIV epidemic, it was anticipated that individuals with both leprosy and HIV would have a higher risk of developing lepromatous disease, experience a faster progression of the disease, and face greater challenges in treatment [9]. To date, there is no evidence to suggest that HIV infection increases susceptibility to leprosy or significantly affects the development of neural or skin lesions associated with the disease [10]. 

In HIV-infected individuals, a paradoxical clinical worsening may occur in preexisting leprosy when an ART-associated reversal reaction (RR) develops. Patients with both leprosy and HIV receiving ART may experience a more severe form of the disease [11].

#### 2.3.2. Clinical Features of *M. leprae* in HIV

Studies have shown that HIV co-infected patients are at an increased risk of developing leprosy reactions after starting ART compared to HIV-negative individuals. For example, a study involving 40 co-infected patients found a significant occurrence of both Type 1 and Type 2 reactions during the study period. Co-infected patients experiencing Type 2 reactions exhibit the classic clinical signs, including widespread painful erythematous nodules on the body, along with fever and arthralgia [12]. Patients with Type 1 reactions may develop erythematous skin lesions, tenderness in the nerves, and potential nerve function impairment [13]. Adjuncts and IRIS-specific management. In HIV–leprosy co-infection, anti-inflammatory adjuncts (e.g., short courses of corticosteroids for reversal or ENL reactions) are central to preserving nerve function, particularly around ART initiation when IRIS may unmask or exacerbate disease. Section 7 details adjunctive strategies and implementation notes specific to PLWH.

### 2.4. Current Standard-of-Care Treatment

#### 2.4.1. Tuberculosis in HIV Co-Infection

Standard of care (TB, PLWH). Drug-susceptible TB is treated with rifamycin-based regimens. ART is initiated early—timing individualized to CD4 count and CNS disease—to balance survival benefit with IRIS risk. Local guideline specifics (drug choices, dosing, extended durations) vary and are not reiterated here; our focus is how these choices interface with ART compatibility and adjunctive/host-directed strategies addressed later.

Adjunctive and host-directed options, with ART/DDI and IRIS considerations specific to PLWH, are detailed in Host-directed and Adjunctive Therapeutics (Section 7).

#### 2.4.2. *Mycobacterium avium* Complex (MAC) Disease in HIV

MAC (PLWH). Standard therapy combines a macrolide + ethambutol (± rifamycin) with durations guided by immune recovery. Durable control depends on effective ART and careful DDI management if rifamycins are used. Detailed dosing follows WHO/NIH guidance and is not reiterated here; our focus is on adjunctive options (e.g., myeloid-supportive measures) and IRIS management, with translational notes in the HDT section (Table 2).

Adjunctive and host-directed options, with ART/DDI and IRIS considerations specific to PLWH, are detailed in Host-directed and Adjunctive Therapeutics (Section 7).

#### 2.4.3. Leprosy (Hansen’s Disease) in HIV

Leprosy (PLWH). Multidrug therapy remains standard; outcomes are strongly influenced by ART-driven immune reconstitution and prevention/management of reversal and ENL reactions. We avoid restating regimen menus; instead, we emphasize adjunctive anti-inflammatory strategies and implementation in HIV care, detailed later (Table 2).

Adjunctive and host-directed options, with ART/DDI and IRIS considerations specific to PLWH, are detailed in Host-directed and Adjunctive Therapeutics (Section 7).

## 3. Drug–Drug Interactions Between Antiretroviral Therapy (ART) and Mycobacterial Medications

Purpose. This section summarizes practical ART–rifamycin compatibility relevant to implementing adjunctive/HDT strategies in PLWH, rather than reproducing full guideline algorithms.

Quick DDI guide (clinically useful highlights): Rifampin is strong inducer; avoid with bictegravir and boosted PIs; dolutegravir typically requires dose adjustment; efavirenz generally compatible. Rifabutin is an alternative when interactions limit rifampin; dose adjustments often needed with boosted ART. Rifapentine’s interaction profile similar to rifampin; check regimen-specific guidance for DTG and TAF use. Boosted regimens (PI/c or EVG/c)—contraindicated with rifampin; consider rifabutin with dose modifications. NRTIs—TDF usually compatible; TAF exposure reduced with rifamycins—consult regimen-specific guidance. Bottom line: if a rifamycin is essential, choose ART for compatibility up front or use rifabutin with adjustments; defer granular dosing to local/WHO/NIH tables.

### 3.1. Rifamycins

Rifamycins (Rifampin, Rifabutin, Rifapentine). Rifampin (RIF) is a potent inducer of hepatic CYP450 enzymes (particularly CYP3A4) and of uridine diphosphate–glucuronosyltransferase (UGT) enzymes [28]. It also induces P-glycoprotein efflux transporters in the gut. As a result, rifampin accelerates the metabolism of many antiretrovirals, dramatically reducing their plasma concentrations. The level of enzyme induction differs among rifamycins: rifampin > rifapentine > rifabutin in relative potency [28]. Rifampin causes the most pronounced interactions and is often contraindicated or not recommended with several ART classes, whereas rifabutin (RFB) has milder effects and is often substituted in HIV patients to allow use of otherwise incompatible ART [14,28]. Rifampin is a potent CYP3A4/UGT1A1 inducer that can reduce PI exposure by up to 95%, whereas rifabutin’s milder induction allows safer use alongside boosted or INSTI-based regimens.

### 3.2. Protease Inhibitors

Protease Inhibitors (PIs): Concomitant rifampin can lower PI levels by 80–95%, essentially contraindicating standard PI-based therapy [15]. For example, rifampin reduces lopinavir, atazanavir, and darunavir exposure to sub-therapeutic levels, even with ritonavir boosting [15]. Simultaneously, PIs (especially ritonavir or cobicistat as boosters) inhibit CYP3A4 and can raise rifampin levels, increasing risk of hepatotoxicity [15]. Due to this bidirectional DDI, rifampin and PIs should not be co-administered. Exceptions are highly experimental, e.g., using super-boosted PI regimens (doubling ritonavir dose) with careful monitoring, but this often causes liver injury and is not a standard practice [15]. Instead, the recommended strategy is to replace rifampin with rifabutin in patients on PI-based ART [15]. Rifabutin has ~80% less enzyme induction effect than rifampin. It can be safely used with PIs if dosed appropriately: because PIs inhibit rifabutin metabolism, the rifabutin dose must be reduced (typically to thrice weekly, instead of daily) [15]. This mitigates toxicity (rifabutin at full dose with a PI would lead to excess rifabutin levels and side effects). Clinical guidelines endorse rifabutin as the preferred rifamycin for TB treatment in patients on PI regimens [15]. If rifabutin is unavailable, an alternative approach is to switch the ART regimen to a non-PI based combination that is compatible with rifampin (see below).

### 3.3. NNRTIs

NNRTIs: The non-nucleoside reverse transcriptase inhibitors vary in how they interact with rifamycins. Efavirenz (EFV) induces CYP3A itself and has a high enough baseline clearance that rifampin’s effect, a ~20–25% reduction in EFV AUC, is usually clinically manageable [15]. Most guidelines allow standard EFV daily with rifampin without dose adjustment, as studies showed adequate EFV levels and viral suppression in patients on concurrent TB therapy [15]. (In some cases, EFV was studied, but no clear benefit over was observed [15].) Thus, EFV-based ART is a preferred option in TB/HIV co-treatment scenarios when rifampin is used, due to its relative safety in co-administration [14]. In contrast, Nevirapine (NVP) levels are significantly lowered by rifampin and vice versa; outcomes with NVP-based ART in TB have been inferior to EFV [15]. If a patient on NVP develops TB, experts recommend switching to EFV or using rifabutin in place of rifampin [15]. Newer NNRTIs are highly susceptible to rifampin induction: Rilpivirine (RPV), Etravirine (ETR), and Doravirine (DOR) are all predominantly metabolized by CYP3A4 and should not be given with rifampin [15]. Rifampin will greatly reduce their concentrations (e.g., >80% reduction), leading to virologic failure [15]. Rifabutin is somewhat gentler: doravirine exposure drops ~50% with rifabutin, but emerging data suggest that doubling the doravirine dose (from to BID) can overcome this induction [15]. This strategy is referenced in DHHS guidelines, but clinical caution is warranted. Overall, EFV is the most rifamycin-friendly NNRTI; others should be avoided or dose-adjusted with rifamycin co-therapy. Delavirdine (an older NNRTI) is a strong CYP3A inhibitor and is contraindicated with any rifamycin due to unpredictable levels [15].

### 3.4. Integrase Strand Transfer Inhibitors (INSTIs)

Integrase Strand Transfer Inhibitors (INSTIs): Rifampin also induces UGT1A1 and CYP3A, which metabolize many INSTIs. Dolutegravir (DTG) and raltegravir (RAL) can be used with rifampin if their doses are increased. Rifampin reduces trough levels of RAL by ~40% and DTG AUC by ~54% [15]. To compensate, RAL dosage is increased from BID to BID, and DTG from daily to BID during rifampin therapy [15]. Clinical trials (e.g., the RIFLAT and INSPIRING studies) have shown that double-dose DTG achieves viral suppression without excess toxicity in TB patients [15]. Therefore, current guidelines include DTG BID as an option for HIV/TB co-treatment (AI). In contrast, bictegravir (BIC) and the boosted INSTI elvitegravir/cobicistat (EVG/c) cannot be safely co-administered with rifampin [15]. Rifampin induction essentially nullifies BIC and EVG levels (they are CYP3A substrates), and therapeutic drug monitoring or dose doubling is not established for these agents [15]. Patients on a BIC-based regimen (e.g., BIC/FTC/TAF) should be switched to an alternative (like DTG or RAL-based) if they require rifampin. The long-acting injectable INSTI cabotegravir (CAB), often given with long-acting RPV, is also incompatible with rifampin/rifapentine—rifamycin induction would likely cause sub-therapeutic CAB/RPV and risk resistance [15]. These long-acting therapies should be avoided in patients who may need rifamycin treatment (e.g., those at high risk for TB). If an INSTI-based ART must be continued with rifampin, DTG or RAL (at adjusted doses as above) are the recommended choices [15]. Rifabutin has minimal effect on INSTI levels: notably, DTG and RAL can be given at their usual doses with rifabutin without adjustment [15]. This makes rifabutin an attractive alternative in patients on integrase-based regimens. For example, a patient on bictegravir could be switched from rifampin to rifabutin for TB treatment, allowing continued use of BIC (with perhaps caution or monitoring). Overall, rifampin necessitates careful selection or dosing of INSTIs, whereas rifabutin offers much more leeway.

Rifapentine: Rifapentine (RPT), used in weekly regimens for latent TB and in some daily short-course regimens, is also a strong inducer of CYP3A (though slightly less than rifampin) [15]. Notably, once-weekly high-dose rifapentine (as in the 12-week latent TB regimen) is not recommended in active TB for HIV patients because it led to unacceptably high relapse rates with rifamycin-resistant TB in studies [15]. Daily rifapentine, as used in the 4-month TB regimen, causes similar interactions as rifampin. That regimen was only studied with EFV-based ART [28], and concurrent use with PIs or other sensitive agents is not advised [14]. If a patient on complex ART is to receive rifapentine (e.g., for latent TB), it is treated akin to rifampin in terms of interaction management. In summary, rifapentine shares rifampin’s liabilities and should be considered incompatible with most PIs, INSTIs, and NNRTIs except EFV. Rifabutin remains the rifamycin of choice for HIV patients on non-EFV ART to minimize DDIs [15].

### 3.5. Macrolides

Macrolides (Clarithromycin and Azithromycin): Macrolides used in MAC treatment also interact with ARVs, though less severely than rifamycins. Clarithromycin is a potent inhibitor of CYP3A4 and is primarily metabolized by CYP3A. Coadministration with ritonavir-boosted PIs can raise clarithromycin levels significantly (two- to three-fold), risking increased macrolide toxicities (e.g., hepatic enzyme elevations, QT prolongation) [4]. In MAC therapy, clarithromycin doses above 1 g/day are contraindicated because high levels have been associated with increased mortality in AIDS patients [4]. When a PI is part of the ART, clinicians have two options: reduce the clarithromycin dose (if the MAC isolate is susceptible and patient can be closely monitored), or more commonly, substitute azithromycin for clarithromycin [4]. Azithromycin is not significantly metabolized by CYP450 and thus has no major interactions with PIs, NNRTIs, or INSTIs [4]. It can be used safely in the presence of these ARVs without dose adjustments. Given its cleaner interaction profile, many experts favor azithromycin as the first-line MAC drug in patients on complex ART regimens, even though clarithromycin has slightly stronger MAC activity. For patients on NNRTIs or unboosted INSTIs, clarithromycin interactions are less profound, but NNRTIs like EFV or ETR may still lower clarithromycin levels somewhat (via induction) and vice versa (clarithromycin can raise EFV levels). These are generally not clinically significant, but therapeutic drug monitoring can be considered in unusual cases. In summary, clarithromycin-PIs is the main problematic combo; switching to azithromycin avoids this DDI entirely [4]. Both macrolides can increase levels of certain concomitant medications (like rifabutin, clarithromycin raises rifabutin AUC, requiring rifabutin dose reduction). Always review each patient’s full med list for other interactions (e.g., clarithromycin with etravirine or maraviroc).

### 3.6. Others

Other TB and leprosy drugs: The remaining first-line TB drugs—isoniazid (INH), pyrazinamide (PZA), ethambutol (EMB)—do not cause major pharmacokinetic interactions with ARVs. Isoniazid can inhibit CYP2C9 and CYP2C19, but this mostly affects drugs like anticonvulsants rather than ARVs [28]. Clinically, INH has not been shown to significantly alter levels of ART drugs, so no dose adjustments are required. However, overlapping toxicities do merit caution: for instance, both INH and some older NRTIs (stavudine, didanosine) can cause peripheral neuropathy, and both PZA and some ARVs (PIs, nevirapine) can cause hepatotoxicity. Close monitoring of liver enzymes is advised when TB therapy and ART are co-administered, as the risk of drug-induced liver injury is additive. Ethambutol and dapsone are chiefly renally excreted and do not affect CYP pathways, posing minimal interaction issues [16]. Clofazimine, used in leprosy and sometimes MAC, is also not known to significantly interact with ARVs; its main concern is additive QT prolongation if paired with other QT-prolonging drugs (certain PIs or INSTIs). In practice, no ART modifications are needed for these agents, but vigilance for side effects is necessary.

### 3.7. Newer and Second-Line Agents

Newer and second-line agents: The advent of bedaquiline and other established but still clinically relevant mycobacterial drugs (e.g., pretomanid, delamanid, linezolid in prolonged MDR-TB regimens) adds new interaction considerations. Bedaquiline (BDQ), now a cornerstone for MDR-TB, is metabolized via CYP3A4. Rifampin induces BDQ metabolism and can lower bedaquiline levels by ~50%, so rifampin should never be given with bedaquiline (patients on MDR-TB therapy do not use rifampin by definition) [4]. Conversely, ritonavir-boosted PIs or potent CYP3A inhibitors could increase bedaquiline levels, raising the risk of BDQ’s dose-dependent QT prolongation [15]. If a patient on PI-based ART requires bedaquiline, expert panels suggest avoiding PIs or switching to an INSTI-based ART during the MDR-TB treatment to minimize cardiac risk (or at least performing ECG monitoring). For example, an HIV/MDR-TB co-infected patient might be managed on dolutegravir-based ART (which has no significant effect on BDQ levels) instead of lopinavir/r. Linezolid, used for TB and atypical mycobacteria, can potentiate neuropathy and bone marrow suppression when given with AZT or d4T, so those combinations should be monitored or avoided [15]. Thalidomide (for severe leprosy ENL reactions) with ART does not have notable kinetic interactions, though both thalidomide and some ARVs are thrombogenic (monitor for DVT). Clinical management strategies, dose adjustments, drug substitutions, or regimen switches—are essential to prevent virologic and mycobacterial treatment failure when rifamycins are required (Table 3).

In all cases of co-administration, the guiding principle is knowing the metabolic profile of each drug and adjusting the regimen to avoid conflicts. Clinicians should reference up-to-date interaction databases (e.g., University of Liverpool HIV/TB drug interaction checker) for nuanced recommendations [15]. As new ARVs (lenacapavir, long-acting injectables) and new anti-TB agents enter use, recommendations will evolve. For instance, the capsid inhibitor lenacapavir is highly long-acting and primarily metabolized by CYP3A; rifampin is expected to significantly reduce its levels, so that combination should be avoided [14]. Likewise, fostemsavir (attachment inhibitor) and otalacapvir (maturation inhibitor in trials) are CYP3A substrates and incompatible with rifampin [14]. On the mycobacterial side, high-dose isoniazid for preventive therapy can be an option if rifamycin interactions preclude use of rifapentine (as per CDC guidelines, 6–9 months of daily INH is an alternative for latent TB in patients on complex ART) [14]. Thus, managing co-infections requires a personalized approach—tailoring the TB/MAC/leprosy regimen and the HIV regimen to work in concert. Clinicians should strive to use an ART regimen that simplifies DDI management: for example, an efavirenz or raltegravir backbone during TB therapy, or a switch to rifabutin when a patient’s ART cannot be changed. With careful selection and dose adjustments, effective concomitant treatment of HIV and mycobacterial disease is achievable without compromising either therapy [15]. As shown in Table 3, doubling dolutegravir or raltegravir doses offsets rifampin-mediated induction, but bictegravir and elvitegravir/cobicistat remain contraindicated.

## 4. Clinical Challenges and Complications

### 4.1. Drug Resistance in Mycobacterial Co-Infections

#### 4.1.1. MDR-TB (Multidrug-Resistant Tuberculosis)

The ongoing spread of drug-resistant TB remains one of the most pressing and challenging issues in global TB control. Patients infected with strains resistant to isoniazid and rifampicin, known as MDR TB, are virtually untreatable with standard first-line therapies. Outbreaks of multidrug-resistant tuberculosis have been associated with poor treatment outcomes and significantly high mortality rates, primarily affecting individuals with advanced HIV infection [17]. A meta-analysis found that HIV-positive individuals had 1.42 times higher odds of developing MDR-TB, a difference that was statistically significant [29]. The treatment of MDR-TB is challenging due to its high pill burden, potential drug interactions, toxicity, and significant cost [18]. Mycobacterial infections stand apart from most bacterial diseases due to the prolonged treatment required to achieve a relapse-free cure with existing therapies. This challenge is even greater in TB, particularly when *M. tuberculosis* is MDR. The World Health Organization (WHO) currently recommends a 20-month treatment regimen for patients with MDR or XDR tuberculosis, using a combination of at least four effective anti-TB drugs. Several second-line TB medications, such as fluoroquinolones, bedaquiline, linezolid, and clofazimine, are metabolized through pathways that may interact with ART drugs [15].

#### 4.1.2. XDR-TB (Extensively Drug-Resistant Tuberculosis)

Extensively drug-resistant tuberculosis (XDR-TB) is a severe form of TB characterized by resistance to multiple key anti-TB drugs. According to the WHO, XDR-TB is defined as TB caused by *Mycobacterium tuberculosis* strains that are resistant to rifampicin, any fluoroquinolone, and at least one additional Group A drug (which includes bedaquiline and linezolid). 

Without appropriate second-line treatment for both tuberculosis and HIV, reported mortality rates for individuals co-infected with XDR TB and HIV can approach 100% [16]. In another study, the key findings included a high mortality rate of 42% and a low rate of successful treatment outcomes (22%) for patients with XDR TB after completing 24 months of treatment in a setting with a high incidence of HIV. All deaths occurred within the first 12 months of treatment. Factors predicting death in this cohort included both tuberculosis-specific (such as TB culture conversion) and HIV-specific (such as ART use) factors [30].

Treatments often require the use of newer or experimental medications, including bedaquiline, linezolid, and pretomanid. However, the safety and efficacy of these drugs have not been fully established, particularly in diverse populations [31].

In high-burden countries, the cost of treating a MDR-TB case is substantially higher than that of drug-susceptible TB, with estimates reaching as much as US$8,365. The expenses for treating XDR-TB are even greater, driven by the need for more costly medications and prolonged treatment periods, placing significant strain on healthcare resources, particularly in low-income settings [19].

#### 4.1.3. MAC Resistance

The treatment and prevention of HIV-associated MAC infection has been enhanced by the use of macrolide antibiotics, such as clarithromycin and azithromycin. However, the effectiveness of these treatments has been somewhat limited by the emergence of macrolide resistance [32]. Resistance to macrolides in MAC is primarily mediated by mutations in the 23S ribosomal RNA gene [33]. Potential risk factors for MAC resistance include macrolide monotherapy, combinations of macrolides with only a quinolone, and macrolide regimens that do not include ethambutol, such as those combining a macrolide with rifampin or rifabutin [34].

The presence of macrolide-resistant MAC strains necessitates modifications to standard treatment protocols. Alternative antibiotics may be required, and combination therapy becomes essential to effectively manage the infection. However, treatment options are limited, and the use of alternative agents may be associated with increased toxicity or drug interactions [35,36,37]. 

## 5. Immune Reconstitution Inflammatory Syndrome (IRIS)

In individuals with HIV, paradoxical reactions following the initiation of antiretroviral therapy (ART) are linked to various underlying infections and are referred to as the IRIS. ART may trigger an intensified granulomatous response that aids in eliminating mycobacterial organisms, but this inflammation can also cause paradoxical harmful reactions characterized by a transient worsening or the emergence of new signs, symptoms, or radiographic manifestations of TB after starting treatment. Importantly, these reactions are not caused by treatment failure or an additional process [38,39].

Studies have symptoms typically appear 4–12 weeks after initiating ART, particularly in patients who experience a substantial increase in CD4+ count from a low baseline, along with successful suppression of HIV replication [40]. While an increase in CD4+ T cell count is often observed after starting ART, it is not a prerequisite for diagnosing IRIS. Clinicians should evaluate other potential causes for the symptoms and perform appropriate assessments to confirm the diagnosis [41]. 

TB-IRIS may present with progressive pulmonary infiltrates or enlarged lymph nodes, potentially mimicking TB treatment failure or relapse [42]. In HIV-positive individuals, MAC-IRIS may present with a range of symptoms, including persistent fever, lymphadenitis, osteomyelitis, granulomatous hepatitis, brain abscesses, and worsening pulmonary infiltrates following ART initiation. Effective management requires continued ART, targeted antimicrobial therapy for MAC, and, in select cases, glucocorticoids to mitigate excessive inflammation [43].

## 6. Side Effects, Overlapping Toxicities, and Adherence Issues

### 6.1. Drug–Drug Interactions and Toxicities

#### 6.1.1. Hepatotoxicity Associated with Rifampin

One of the major challenges in the management of HIV and mycobacterial co-infections is the overlapping toxicities of ART and anti-mycobacterial therapies. Rifampin, a key component of TB treatment, has been associated with liver toxicity. Its hepatotoxic effects are driven by mechanisms such as oxidative stress and immune-mediated reactions. Clinical presentations vary from mild liver enzyme elevations to severe hepatitis, and in rare cases, acute liver failure [44]. Rifampin is also known for causing significant drug interactions and is often contraindicated or not recommended with several ART classes. When used together, rifampin can reduce protease inhibitor (PI) levels by 80–95%, making standard PI-based therapy essentially ineffective [5].

#### 6.1.2. Nephrotoxicity and Ototoxicity of Second-Line Anti-TB Drugs

Second-line injectable anti-TB drugs, including aminoglycosides (such as amikacin and kanamycin) and capreomycin, are linked to dose-dependent nephrotoxicity and ototoxicity. Research has shown that these drugs can cause kidney dysfunction and hearing loss in patients with MDR-TB [45].

#### 6.1.3. Impact of ART on Ototoxicity

The risk of ototoxicity is heightened in patients co-infected with HIV and TB. HIV infection has been recognized as a significant risk factor for developing hearing loss during anti-TB treatment [7].

#### 6.1.4. Adherence Issues

The treatment of TB, MAC, and leprosy in HIV-positive individuals is frequently challenging due to difficulties in adhering to lengthy and intricate therapeutic regimens. The requirement to take multiple drugs, often with overlapping toxicities and specific dietary or timing constraints, presents substantial barriers to maintaining treatment adherence [46,47].

Poor adherence to ART and anti-TB therapy significantly contributes to treatment failure, hindering efforts to control and prevent these diseases. Non-compliance can increase the risk of drug resistance, prolong infectiousness, lead to TB relapse, and, in severe cases, result in death [48].

Research has shown that a high pill burden adversely impacts patient adherence. For example, a study on diabetes patients found that those receiving a fixed-dose combination had a 12.8% higher adherence rate than those taking multiple individual medications. Additionally, a meta-analysis revealed a 26% decrease in non-compliance when fixed-dose combinations were used for conditions such as hypertension, HIV, and TB [49]. Furthermore, hemodialysis patients with a higher pill burden reported experiencing a greater overall treatment burden than those with fewer medications, highlighting the broader challenges associated with complex drug regimens [50].

## 7. Host-Directed and Adjunctive Therapeutics

This section prioritizes host-directed and adjunctive strategies with HIV-specific translational considerations—ART compatibility, IRIS risk, and immune recovery—rather than reiterating standard antimicrobial algorithms. We incorporate non-tuberculous mycobacterial disease (MAC) and leprosy where data exist, areas less developed in recent TB-centric reviews. Implementation notes for feasibility in PLWH are emphasized throughout.

### 7.1. Host-Directed Therapies (HDTs)

HDT is a promising and evolving strategy in anti-infective treatment. It aims to modulate host cell factors essential for pathogen replication or persistence, strengthen protective immune responses, mitigate excessive inflammation, and restore immune balance at sites of infection [51]. Implementation in PLWH (summary): ART compatibility noted; IRIS considerations addressed where relevant; feasibility and monitoring signals summarized above.

#### 7.1.1. Vitamin D Supplementation

**Figure 1:**
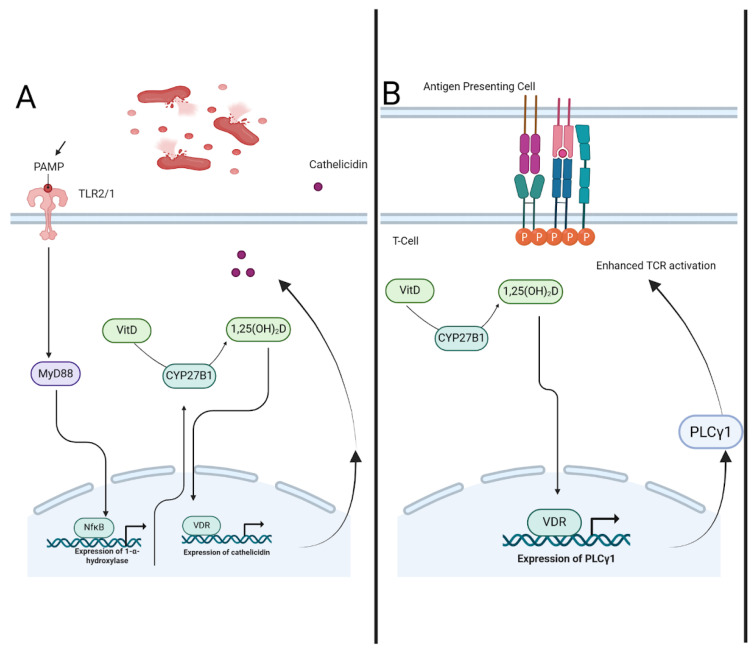
Immunomodulatory role of vitamin D in innate and adaptive immune responses against *Mycobacterium tuberculosis*. (**A**) Upon recognition of pathogen-associated molecular patterns (PAMPs) by TLR2/1 on macrophages, MyD88 signaling induces CYP27B1 expression, converting inactive vitamin D (VitD) into its active form 1,25(OH)_2_D. This active metabolite binds to the VDR, promoting the transcription of cathelicidin, an antimicrobial peptide crucial for innate defense. (**B**) In adaptive immunity, 1,25(OH)_2_D binds VDR in T cells, upregulating phospholipase C gamma 1 (PLCγ1) expression. This enhances T-cell receptor (TCR) activation, boosting antigen-specific responses. Created in BioRender. Sasaninia, K. (2025). https://BioRender.com/bjxhn96 (accessed on 3 July 2025).

Vitamin D (Vit-D) supplementation has shown potential in inhibiting both HIV replication and *Mycobacterium tuberculosis* growth. A key mechanism involves cathelicidin, an antimicrobial peptide crucial to the innate immune response against TB. When *M. tuberculosis* binds to toll-like receptors (TLR2/1) on macrophages, it upregulates 1α-hydroxylase expression, leading to the conversion of Vit-D into its active form, 1.25(OH)_2_D. Vitamin D plays a dual role in enhancing both innate and adaptive immunity against *Mycobacterium tuberculosis* through distinct yet complementary mechanisms (Figure 1). This active metabolite binds to the vitamin D receptor (VDR), triggering cathelicidin production and enhancing antimicrobial activity. Additionally, Vit-D promotes adaptive immunity by activating T helper cells through VDR-mediated signaling, significantly increasing PLC-γ1 expression (by 75-fold) to enhance T-cell receptor (TCR) activation [45]. In macrophages, activation of TLR2/1 signaling by *M. tuberculosis* triggers the MyD88 pathway, leading to conversion of vitamin D into its active form and upregulation of cathelicidin, a key antimicrobial peptide (Figure 1A).

The inclusion of Vit-D in the treatment of moderately advanced pulmonary tuberculosis (PTB) and HIV has demonstrated a significant impact on sputum conversion rates compared to placebo. A study conducted in Jakarta reported that patients receiving Vit-D showed higher sputum conversion and radiological improvement (100%) compared to the placebo group (76.7%), a difference that was statistically significant [52]. In T cells, 1,25(OH)_2_D promotes transcription of PLCγ1 via VDR signaling, enhancing T-cell receptor sensitivity and adaptive immune activation (Figure 1B). Implementation in PLWH (summary): ART compatibility noted; IRIS considerations addressed where relevant; feasibility and monitoring signals summarized above.

#### 7.1.2. Metformin

**Figure 2:**
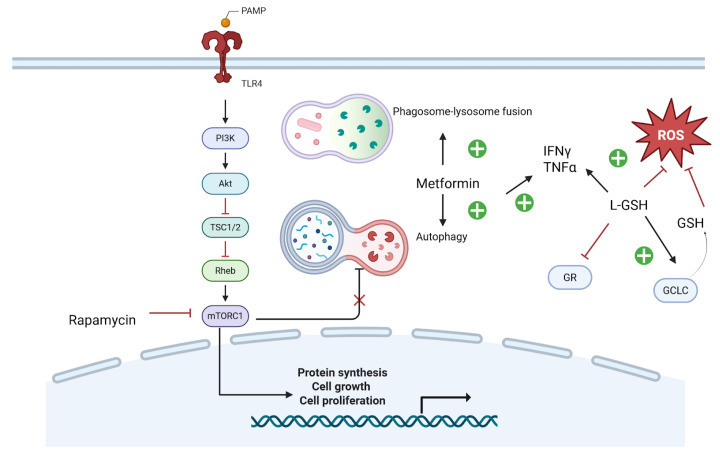
Host-directed therapeutic mechanisms of metformin, rapamycin, and glutathione (GSH) in enhancing immunity against *Mycobacterium tuberculosis*. Positive signs (+) indicate enhancement, Flat head arrows indicate inhibition. Crosses (x) over flat head arrows indicate loss of inhibition. Metformin promotes phagosome–lysosome fusion and autophagy, enhancing intracellular bacterial clearance. Rapamycin inhibits the mTORC1 pathway, suppressing protein synthesis and promoting autophagy via TSC1/2–Rheb signaling. Glutathione (GSH), through its reduced (L-GSH) and oxidized forms, modulates oxidative stress by scavenging reactive oxygen species (ROS), supported by enzymes like GCLC and GR. Together, these agents amplify IFN-γ and TNF-α mediated immune responses and regulate redox balance to contain infection. Created in BioRender. Sasaninia, K. (2025). https://BioRender.com/7ss6pc1 (accessed on 3 July 2025).

Metformin (MET), an FDA-approved drug widely used for managing type 2 diabetes (T2D), has shown promising immunomodulatory effects in TB [53]. Research indicates that MET enhances intracellular phagosome–lysosome fusion and induces autophagy in *M. tuberculosis* (Mtb)-infected macrophages [54]. Studies further demonstrate that MET promotes macrophage autophagy, leading to improved bacterial clearance or containment, while also modulating inflammation through elevated levels of TNF-α, interferon-γ, and interleukin-1β. Metformin enhances phagosome–lysosome fusion and stimulates autophagy, while rapamycin suppresses mTORC1, reducing cellular proliferation and redirecting resources toward immune activation (Figure 2). By activating both innate and adaptive immune responses, MET as a HDT could potentially shorten TB treatment duration, enhance treatment outcomes, accelerate bacterial eradication, and reduce TB transmission [49,50]. HDTs such as metformin and rapamycin have shown promising roles in enhancing autophagy and modulating intracellular signaling pathways to improve *M. tuberculosis* clearance (Figure 2). Implementation in PLWH (summary): ART compatibility noted; IRIS considerations addressed where relevant; feasibility and monitoring signals summarized above.

#### 7.1.3. Glutathione

Glutathione (GSH) holds significant therapeutic potential in improving treatment outcomes for TB-HIV co-infections by enhancing antibiotic efficacy, modulating inflammation, and restoring immune function [55]. As a critical component of innate immunity against M. tuberculosis*(Mtb), GSH plays a vital role in controlling infection. However, HIV infection leads to depleted GSH levels, compromising the immune system’s ability to combat Mtb [56]. This altered environment facilitates microbial persistence and reduces the efficacy of antibiotics and antiretrovirals [55]. GSH, a key antioxidant, supports immune responses by reducing ROS levels and restoring redox homeostasis through enzymes like glutamate-cysteine ligase (GCLC) and glutathione reductase (GR), both of which are essential in the defense against mycobacterial infection (Figure 2).

The link between HIV-induced GSH depletion and TB pathogenesis underscores its potential as an adjunctive therapy, offering a means to strengthen existing treatment regimens while simultaneously reducing inflammation and immune dysfunction [56].

Research indicates that restoring GSH levels in HIV-positive patients can reverse the impairment of innate immune functions in macrophages. Supplementation of GSH has been shown to reduce reactive oxygen species (ROS) production and modulate key antioxidant enzymes, with an increase in glutamate-cysteine ligase catalytic subunit (GCLC) and a decrease in glutathione reductase (GSR). These findings suggest that GSH supplementation helps mitigate oxidative stress, thereby improving immune function and enhancing the body’s ability to combat infections like *M. tuberculosis* in co-infected individuals [57]. Implementation in PLWH (summary): ART compatibility noted; IRIS considerations addressed where relevant; feasibility and monitoring signals summarized above.

#### 7.1.4. mTOR Inhibitors

A study using C3HeB/FeJ mice infected with *Mycobacterium tuberculosis* demonstrated that rapamycin administration, either alone or in combination with moxifloxacin, reduced lung inflammation and decreased both the number and size of caseating necrotic granulomas. These findings highlight rapamycin’s potential as an adjunctive therapy for tuberculosis, offering immunomodulatory benefits that may enhance treatment outcomes by regulating the host immune response [58,59]. The mechanistic target of rapamycin (mTOR) pathway is pivotal in regulating autophagy, a cellular process vital for degrading intracellular pathogens such as *Mycobacterium tuberculosis*. Inhibition of mTOR by rapamycin enhances autophagic activity, which improves the clearance of mycobacteria within host cells. This mechanism underscores rapamycin’s potential as an adjunctive therapy in tuberculosis treatment, facilitating more efficient elimination of the pathogen through enhanced autophagy [60]. These findings support the integration of HDTs alongside conventional antimicrobial therapy to improve outcomes in TB-HIV co-infected patients (Figure 2). Implementation in PLWH (summary): ART compatibility noted; IRIS considerations addressed where relevant; feasibility and monitoring signals summarized above.

#### 7.1.5. Anti-Inflammatory Agents

Nonsteroidal anti-inflammatory drugs (NSAIDs) and corticosteroids have been studied as supplementary treatments to reduce excessive inflammation in TB and MAC infections. Corticosteroids are frequently employed to treat severe forms of tuberculosis, including miliary TB, respiratory failure, central nervous system complications, and pericarditis. The World Health Organization advises the use of adjuvant corticosteroid therapy with dexamethasone or prednisolone, gradually tapered over 6–8 weeks, for individuals with tuberculous meningitis [20]. However, corticosteroid use should be approached with caution, as it can increase the risk of other infections and interact with anti-TB medications, potentially influencing treatment outcomes [61]. Implementation in PLWH (summary): ART compatibility noted; IRIS considerations addressed where relevant; feasibility and monitoring signals summarized above.

### 7.2. Immunotherapies and Cytokine Modulators

#### 7.2.1. IFN-γ

Multiple studies have explored the effectiveness of IFN-γ in treating refractory tuberculosis. A report on two patients with refractory central nervous system TB showed substantial improvement after adding adjuvant IFN-γ therapy, with both patients exhibiting significant clinical and radiological improvements [62].

The role of IFN-γ in treating MAC infections has also been examined. In a case involving a patient with complete IFN-γ receptor deficiency and disseminated MAC infection, adjunctive IFN-α2b therapy effectively reduced hepatosplenomegaly and improved clinical outcomes [63]. Furthermore, studies suggest that IFN-γ therapy can enhance antimicrobial treatment for MAC infections, especially in patients with certain immunodeficiencies [64].

Although IFN-γ therapy shows potential, its use must be carefully evaluated. A randomized controlled trial examining adjunctive IFN-γ in drug-susceptible pulmonary TB found no significant therapeutic benefit. As such, the decision to administer IFN-γ should be personalized, considering the patient’s specific condition and immune status [61]. Implementation in PLWH (summary): ART compatibility noted; IRIS considerations addressed where relevant; feasibility and monitoring signals summarized above.

#### 7.2.2. Granulocyte-Macrophage Colony-Stimulating Factor (GM-CSF)

Recombinant human colony-stimulating factors, including granulocyte colony-stimulating factor (G-CSF) and granulocyte-macrophage colony-stimulating factor (GM-CSF), are hematopoietic cytokines that stimulate neutrophil production and enhance their functional capacity [65]. In vitro studies indicate that GM-CSF can enhance the antiviral activity of zidovudine (AZT) in macrophages, suggesting its potential role in improving viral reservoir clearance when combined with ART [66].

HIV infection profoundly impacts cytokine regulation, significantly reducing the production of GM-CSF and other essential immune mediators [67]. Multiple studies have demonstrated that GM-CSF positively impacts key aspects of HIV infection, including reductions in plasma HIV RNA levels and increases in CD4+ lymphocyte counts [68]. In HIV-positive individuals, immune suppression often leads to opportunistic infections such as MAC. A case study reported the successful resolution of treatment-resistant MAC disease following the addition of GM-CSF, underscoring its potential role as an adjunctive immunomodulatory therapy in difficult-to-treat cases [69].

Research has shown that the diminished production of GM-CSF by HIV-infected macrophages compromises their ability to control MTB infection [70]. GM-CSF plays a key role in the proper development and function of alveolar macrophages, which are essential for the body’s defense against infections and the regulation of inflammatory responses [42]. Implementation in PLWH (summary): ART compatibility noted; IRIS considerations addressed where relevant; feasibility and monitoring signals summarized above.

#### 7.2.3. Interleukin-7 

Interleukin-7 (IL-7) is an antiapoptotic cytokine belonging to the common γ-chain family, playing a crucial role in the proliferation and survival of lymphocytes. Studies have demonstrated that IL-7 prevents lymphocyte apoptosis, restores the function of CD4+ and CD8+ T cells, and enhances survival in animal models of bacterial and fungal sepsis [71]. In individuals with HIV, IL-7 levels are frequently elevated, especially when CD4+ T-cell counts are low, likely as a compensatory mechanism to counteract lymphopenia [72].

Research has explored the potential of IL-7 in reducing the severity of IRIS [73]. However, the relationship between IL-7 and IRIS is complex and not fully understood. Some studies suggest that persistently elevated IL-7 levels, even after ART-induced CD4+ T-cell recovery, may contribute to the inflammatory responses that characterize IRIS [8]. This suggests that while IL-7 supports T-cell proliferation, its exact role in IRIS remains unclear and requires further investigation. Implementation in PLWH (summary): ART compatibility noted; IRIS considerations addressed where relevant; feasibility and monitoring signals summarized above.

### 7.3. Novel Antimicrobials

Drug-resistant mycobacteria can now be effectively combatted with novel antimicrobials because of developments in molecular drug development.

#### 7.3.1. Bedaquiline 

The antimycobacterial drug bedaquiline is a diarylquinoline that works by blocking the enzyme mycobacterial ATP synthase, which is necessary for *Mycobacterium tuberculosis* to produce energy. Cell death results from this inhibition’s disruption of the bacterium’s energy metabolism [21]. Bedaquiline has been shown in clinical trials to be effective in treating MDR-TB. According to a systematic review and meta-analysis, patients with MDR-TB had better results when bedaquiline was included in their treatment plans [21]. Furthermore, bedaquiline has demonstrated efficacy in treating XDR-TB, providing a useful alternative in situations where traditional therapies are not working [74].

Even though bedaquiline is a major breakthrough in the fight against drug-resistant tuberculosis, its use needs to be carefully monitored to avoid resistance developing. The atpE gene, which codes for an ATP synthase component, has been linked to reduced bedaquiline susceptibility. Bedaquiline should therefore be used in combination therapy under close medical monitoring in order to maximize treatment results and prevent the emergence of resistance [13,21,75].

#### 7.3.2. Pretomanid

Pretomanid, a nitroimidazooxazine molecule, provides a shorter and more efficient treatment course for drug-resistant TB when used in combination regimens [76]. Pretomanid tablets were approved by the U.S. FDA in August 2019 for the treatment of adults with treatment-intolerant or nonresponsive MDR-TB or pulmonary XDR-TB when used in conjunction with bedaquiline and linezolid. The approval was granted following clinical trials that showcased the effectiveness of the BPaL regimen (bedaquiline, pretomanid, and linezolid). The key Nix-TB trial demonstrated that this all-oral regimen led to successful outcomes in a substantial number of patients with highly drug-resistant TB, providing a shorter six-month treatment duration compared to conventional therapies [76,77]. Pretomanid exerts its antimicrobial effects against *Mycobacterium tuberculosis* by blocking mycolic acid biosynthesis, a crucial component of the bacterial cell wall. Additionally, under anaerobic conditions, it generates reactive nitrogen species, enhancing its bactericidal activity against both actively replicating and dormant bacteria [78]. Although pretomanid has demonstrated efficacy in treating drug-resistant TB, its administration requires careful monitoring due to possible adverse effects and the importance of strict adherence to the treatment regimen. Healthcare providers should assess its risks and benefits on a case-by-case basis, taking into account potential drug interactions and patient-specific tolerability.

#### 7.3.3. Delamanid 

Clinical trials have examined the effectiveness of delamanid when used alongside optimized background regimens (OBR) for MDR-TB. A phase 3 study evaluated its safety and efficacy during the first six months of treatment, showing that regimens including delamanid were linked to improved treatment outcomes [79].

Additionally, a retrospective national cohort study in Latvia found that among 19 patients who completed treatment with delamanid, 89.5% had pre-XDR or XDR-TB, highlighting delamanid’s potential efficacy in managing severe drug-resistant TB cases [80].

**Figure 3:**
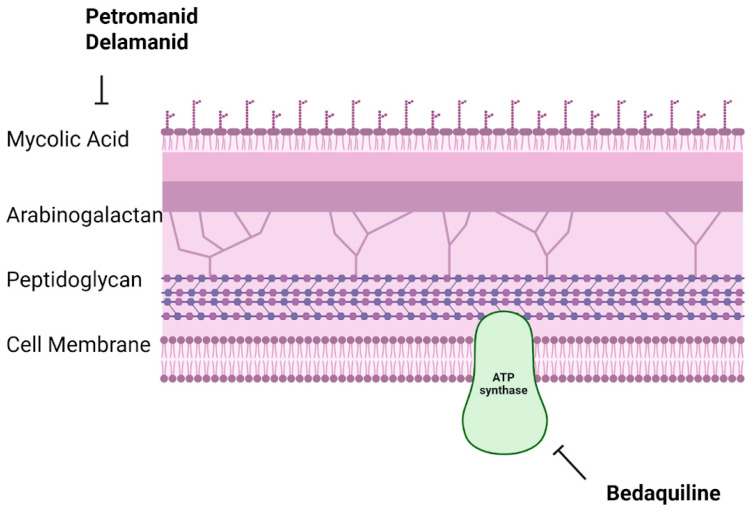
Mechanisms of action of novel antimycobacterial drugs Pretomanid, Delamanid, and Bedaquiline against *Mycobacterium tuberculosis*. Pretomanid and Delamanid inhibit mycolic acid biosynthesis, disrupting cell wall integrity, whereas Bedaquiline inhibits ATP synthase, impairing bacterial energy metabolism. Created in BioRender. Sasaninia K. (2025). https://BioRender.com/69aaroa (accessed on 3 July 2025).

The WHO has released a position statement on delamanid for MDR-TB, recognizing its potential advantages and offering recommendations for its inclusion in treatment regimens. Novel therapeutics like Pretomanid and Delamanid disrupt *Mycobacterium tuberculosis* cell wall synthesis by inhibiting mycolic acid production, while Bedaquiline targets ATP synthase, disrupting bacterial energy production (Figure 3). These mechanisms highlight promising approaches to overcoming drug-resistant tuberculosis through targeted disruption of essential bacterial functions (Figure 3).

### 7.4. Vaccines and Immunotherapy Developments

One of the main areas of study continues to be the development of efficient vaccines against TB and other mycobacterial diseases in HIV:

#### 7.4.1. MTBVAC

MTBVAC, created by Professor Carlos Martín and his research team at the University of Zaragoza in Spain, is the sole live-attenuated M. tuberculosis-based vaccine currently under clinical development. Its development focuses on offering improved protection against respiratory forms of tuberculosis, surpassing the effectiveness of the existing BCG vaccine [81,82].

Considering the significant co-occurrence of TB and HIV, it is essential to assess the effectiveness of MTBVAC in HIV-positive populations. Early-phase clinical trials and preclinical studies have shown that MTBVAC is safe and stimulates strong immune responses in individuals with HIV, indicating its potential advantages for this group.

#### 7.4.2. VPM1002

VPM1002 is a recombinant Bacillus Calmette–Guérin (BCG) vaccine created to boost immune responses, especially in immunocompromised groups, including those infected with HIV [83]. VPM1002 was developed by substituting the urease C gene in BCG with the listeriolysin (Hly) gene from *Listeria monocytogenes*. This alteration enables the vaccine to break down the phagosome, enhancing antigen presentation through MHC-I pathways and stimulating strong CD8+ T-cell responses [84]. Although detailed data on VPM1002′s effectiveness in HIV-infected adults are scarce, its design indicates promising potential. The vaccine’s capacity to boost MHC-I-mediated immune responses may provide enhanced protection for immunocompromised individuals, who are more susceptible to TB [84].

#### 7.4.3. Therapeutic Vaccines

Therapeutic vaccines are designed to stimulate or strengthen the immune response to modify the progression of a disease in patients. These vaccines have the potential to offer long-lasting, non-invasive, and cost-effective treatment options for a large population of HIV-infected individuals [85]. An additional definition of therapeutic vaccination in the context of tuberculosis is the administration of a vaccine to individuals with signs of *M. tuberculosis* exposure, such as a positive interferon-gamma release assay (IGRA + ve), to prevent the progression to active tuberculosis. The WHO suggests that therapeutic vaccines should be targeted at all TB patients, irrespective of age, drug sensitivity, or comorbidities [86]. Therapeutic TB vaccines currently in clinical development are classified into several categories: whole killed and fragmented-cell vaccines, live attenuated vaccines, adjuvanted protein subunit vaccines, and viral vectored vaccines [86].

Although therapeutic vaccines targeting *Mycobacterium avium* complex (MAC) are not as advanced as those for MTB, research in this field is ongoing. One study explored the prophylactic effectiveness of two different vaccine delivery platforms against *Mycobacterium avium*, showing promising potential for future vaccine development [87].

#### 7.4.4. Deregulated Host Transcription Factors as Therapeutic Targets in HIV-Mycobacterial Coinfection

A novel angle in host-directed therapy is the manipulation of host cell transcription factors that are abnormally regulated during HIV/mycobacterial co-infection. Chronic infection and HIV-induced immune dysfunction can skew the activity of certain transcription factors (TFs), thereby impairing immune responses or exacerbating pathology. Targeting these “deregulated” TFs offers a strategic way to recalibrate the host environment in favor of pathogen clearance. One prominent example is hypoxia-inducible factor 1α (HIF-1α). HIF-1α is normally stabilized in hypoxic tissues and drives a potent antimicrobial responseupregulating glycolysis, tumor necrosis factor, nitric oxide, and other host-protective mediators essential for containing *M. tuberculosis* [88]. HIV and other co-morbidities (e.g., diabetes) can impair HIF-1α responses, leading to suboptimal macrophage killing. Boosting HIF-1α activity pharmacologically is therefore an attractive strategy. Interestingly, the antidiabetic drug metformin activates AMPK and mildly inhibits mitochondrial respiration, which indirectly stabilizes HIF-1α in myeloid cells. This contributes to metformin’s observed enhancement of TB control in experimental models [54]. Future therapies might include direct HIF-1α stimulators (prolyl hydroxylase inhibitors currently used for anemia) to reinforce this pathway and turbocharge the host’s bactericidal capacity [54].

Another key factor is NF-κB, a master regulator of inflammation and cell survival. In the context of HIV-TB co-infection, NF-κB’s role is double-edged. On one hand, NF-κB activation in macrophages and T cells is crucial for producing cytokines like IL-12 and TNF-α that activate antimycobacterial defenses. On the other hand, HIV can hijack NF-κB, the HIV long terminal repeat (LTR) contains NF-κB binding sites, so active NF-κB can enhance HIV replication. Moreover, excessive or unchecked NF-κB activity contributes to immunopathology, driving the formation of destructive granulomas and systemic inflammation [89]. Therapeutically modulating NF-κB requires nuance: partial inhibition might quell harmful inflammation (and HIV replication) without crippling host immunity. One approach under investigation is the inhibition of PARP1, a nuclear co-factor required for full NF-κB transcriptional activity. In a recent review, PARP1 was highlighted as a driver of persistent NF-κB-mediated lung inflammation in TB; its inhibition could reduce lung tissue damage [89]. Adjunctive agents like aspirin (which inhibits IκB kinase to some extent) or corticosteroids (which broadly suppress NF-κB activity) have already shown benefits in reducing TB inflammation and IRIS severity, respectively. Ongoing research aims to develop more targeted NF-κB pathway inhibitors (or pathway reseters) that could be given transiently during acute phases to prevent immunopathology without increasing risk of infection.

HIV and mycobacterial infections can also dysregulate NFAT (nuclear factor of activated T-cells) family transcription factors. Notably, NFAT5—which responds to osmotic stress and cytokine signals—was found to be overactivated in macrophages during TB/HIV co-infection. This has consequences: a 2012 study showed that *M. tuberculosis* infection of macrophages induced NFAT5, which in turn upregulated HIV-1 transcription, fueling viral replication [90]. By silencing NFAT5, researchers inhibited TB-induced HIV replication in co-infected cells [90]. NFAT5 thus represents a potential target to break the deleterious cycle whereby TB amplifies HIV via host signaling. An NFAT5 inhibitor or knockdown (if achievable in vivo) might suppress HIV rebound during TB episodes, helping to maintain viral suppression. This is a prime example of targeting a host factor to indirectly counteract a pathogen–pathogen interaction (TB’s effect on HIV).

The master regulators of autophagy and metabolism are also of great interest. The transcription factor EB (TFEB), which controls lysosomal biogenesis and autophagy gene networks, is often kept inactive by mTOR signaling during steady-state conditions. In chronic HIV infection, monocytes and macrophages can have functional autophagy defects. Activating TFEB can override these defects by boosting the cell’s lysosomal degradation capacity. Bryk et al. recently discovered a small molecule (termed “Compound 2062”) that activates TFEB; when applied to *M. tuberculosis*-infected macrophages, it enhanced bacterial clearance and synergized with rifampin [91]. In vivo, TFEB activation reduced lung mycobacterial burden and pathology in mice [91]. These findings underscore TFEB as a druggable host target—a selective TFEB agonist could serve as an adjunct to TB antibiotics, especially valuable in patients whose immune cells are impaired by HIV. Along similar lines, factors like Nrf2 (nuclear factor erythroid 2-related factor 2), which governs the antioxidant response, are being studied. Chronic HIV and TB infections generate oxidative stress that can damage host tissues and dampen immunity. Nrf2 is often suppressed by the pathogen’s manipulation or by HIV proteins, leading to excessive inflammation and tissue injury. Compounds that activate Nrf2 (such as dimethyl fumarate or sulforaphane) could restore redox balance and protect tissue during TB treatment. Indeed, activation of Nrf2 via the autophagy adaptor p62 has been proposed as one mechanism by which metformin confers benefit [92]. Though not yet tested in clinical trials for TB, Nrf2 inducers are an intriguing area for future host-directed interventions to reduce TB-associated lung damage.

In conclusion, manipulating host transcriptional programs represents a cutting-edge approach to combat HIV-associated mycobacterial diseases. These strategies do not replace antibiotics or antiretrovirals; rather, they complement them by correcting the underlying host deficits that allow pathogens to proliferate. By targeting deregulated TFs like HIF-1α, NF-κB, NFAT5, and TFEB, we can potentially re-tune the immune system: enhancing bacterial killing, limiting harmful inflammation, and even suppressing HIV’s exploitation of host pathways. Challenges remain—specificity is crucial (global suppression of NF-κB, for example, could be deleterious), and any host-targeted therapy must be carefully balanced to avoid tipping into immunosuppression. Nonetheless, early-stage research and translational studies are encouraging. The paradigm is shifting from a purely pathogen-centric view to one that equally considers empowering the host. As our understanding of host–pathogen interactions deepens, we anticipate new adjunctive therapies that will synergize with existing drugs, leading to shorter, safer treatment courses for patients with HIV-TB, HIV-MAC, and other co-infections. In an era of rising resistance and persistent co-morbidity, targeting the host’s own regulatory mechanisms offers a promising avenue to stay one step ahead of these formidable pathogens [88].

### 7.5. Recent Clinical Trials and Future Directions

Treating TB and MAC infections in individuals with HIV presents significant challenges. However, recent progress in clinical research offers hope for enhancing patient outcomes. Current advancements emphasize the reduction in treatment duration, the exploration of host-targeted therapeutic strategies, and the potential incorporation of new vaccine candidates into existing treatment approaches. 

#### 7.5.1. Shortening TB and MAC Therapy Regimens

Incomplete TB treatment contributes to the emergence of drug-resistant strains, complicating future management [93,94]. While efforts to shorten DS-TB therapy have progressed, the pace has been slow, with treatment duration decreasing from 24 months in 1952 to 4 months in 2022. A key milestone in this advancement was Study 31/A5349, a multicenter Phase III trial, which successfully demonstrated that a 4-month regimen using rifapentine and moxifloxacin was as effective as the standard 6-month course, marking a significant step toward improving treatment adherence and outcomes [94]. In contrast, advancements in MDR- and XDR-TB treatment have led to the development of shorter, more effective regimens that utilize combinations of bedaquiline, pretomanid, and linezolid, offering improved outcomes and treatment feasibility [22].

For MAC infections, ongoing trials are assessing the efficacy of shorter treatment regimens incorporating newer agents such as linezolid and clofazimine. Preliminary findings indicate potential benefits, including enhanced treatment success and fewer adverse effects [95]. A randomized clinical trial evaluated the safety and effectiveness of substituting rifampicin with clofazimine in the standard MAC-PD treatment regimen. The study compared a clofazimine-ethambutol-macrolide regimen to the conventional rifampicin-ethambutol-macrolide approach. Findings demonstrated that the clofazimine-based regimen achieved similar culture conversion rates, indicating its potential as an alternative to rifampicin in MAC-PD therapy [96]. Studies on linezolid for MAC-PD remain limited. However, a retrospective analysis assessed its use in patients with refractory MAC lung disease who received linezolid as part of their treatment regimen. The findings suggested that linezolid may offer some therapeutic benefit for individuals unresponsive to conventional therapies [95]. 

#### 7.5.2. Innovative Host-Directed Therapies and Immunotherapy Trials

Alongside the development of novel antimicrobial strategies, there has been a significant increase in the investigation of HDTs and immunotherapies. These approaches aim to enhance the host immune response to infections while minimizing the harmful effects of inflammation. Ongoing clinical trials are evaluating the efficacy of immune modulators such as IFN-γ, GM-CSF, and mTOR inhibitors, with the goal of improving macrophage functionality and facilitating mycobacterial clearance [62,63,64,66,67,69]. Additionally, adjunctive treatments, including antioxidants like glutathione and anti-inflammatory agents, are being explored to mitigate immune-mediated tissue damage in patients with TB and MAC co-infections [57,58,59]. Preliminary studies have shown promising outcomes, indicating that HDTs may not only complement antimicrobial treatments but also reduce the duration and severity of infections.

#### 7.5.3. Integrated Models of TB and HIV Care

In response to the significant challenges posed by TB-HIV co-morbidity, the WHO has recommended integrating TB and HIV services at least at the facility level [97]. This has led to the implementation of various models, including linkage, collaboration, and full integration, each facing unique challenges in different settings. For example, in a linkage model, patients diagnosed with either infection are referred to another facility or unit for testing and treatment of the other infection. In contrast, a fully integrated model provides both TB and HIV services within a single facility, with the same healthcare providers delivering care for both conditions. A substantial body of evidence indicates that the fully integrated model offers the most significant benefits for patients, healthcare systems, and providers, making it the preferred approach for managing TB-HIV co-infection [98].

The optimal timing for initiating ART in patients co-infected with TB and HIV has been widely studied. A meta-analysis evaluated the impact of starting ART early, within 2–4 weeks of beginning TB treatment, on various treatment outcomes. The results indicated that early ART initiation was linked to a reduction in all-cause mortality [23]. In contrast, another study found that starting ART early during TB treatment did not show significant benefits compared to delaying its initiation in terms of TB treatment failure, recurrence, or mortality. This implies that in some cases, ART may be postponed until after the completion of TB treatment [24].

#### 7.5.4. Potential Role of Novel Vaccine Candidates (e.g., M72/AS01E)

The M72/AS01E vaccine candidate has shown potential in TB prevention. A Phase 2b clinical trial conducted in Southern and Eastern Africa enrolled 3,573 HIV-negative adults aged 18–50 years with latent *Mycobacterium tuberculosis* infection [99].

Participants were administered two doses of either M72/AS01E or a placebo, spaced one month apart. Over a three-year follow-up period, the vaccine demonstrated a 54% efficacy in preventing the progression to active pulmonary TB.

Expanding on previous research, the Bill & Melinda Gates Medical Research Institute launched a Phase 3 clinical trial in March 2024 to further investigate the efficacy and safety of M72/AS01E. This study aims to encompass a diverse participant pool, including individuals with HIV, to assess the vaccine’s protective effects across various demographic groups [99,100].

Implementation in PLWH (at a glance)

- Vitamin D—ART compatibility: none expected; IRIS: neutral; Feasibility/monitoring: inexpensive, monitor calcium/renal function if high-dose.
- Metformin—ART compatibility: generally compatible; IRIS: potential anti-inflammatory benefit; Feasibility/monitoring: watch renal function and GI tolerance.
- mTOR modulation—ART compatibility: check DDIs (CYP3A, P-gp); IRIS: may blunt hyper-inflammation; Feasibility/monitoring: drug levels/toxicity if applicable.
- Glutathione augmentation—ART compatibility: none expected; IRIS: may reduce oxidative injury; Feasibility/monitoring: formulation access, hepatic/renal status.
- Cytokines (IFN-γ, GM-CSF)—ART compatibility: none direct; IRIS: use cautiously in high-inflammation states; Feasibility/monitoring: hematologic/immune monitoring.
- Therapeutic vaccines—ART compatibility: none direct; IRIS: consider timing around ART initiation; Feasibility/monitoring: trial availability, immunogenicity readouts.

## 8. Discussion

Patients with HIV-associated mycobacterial infections face overlapping therapeutic challenges, but advances in both antimicrobials and host-directed strategies are reshaping outcomes. Drug–drug interactions remain central obstacles: rifampicin, widely used in drug-susceptible TB, induces cytochrome P450 enzymes and markedly reduces plasma concentrations of protease inhibitors and some integrase inhibitors. Rifabutin is often substituted, or ART regimens are adjusted (e.g., dose modification of dolutegravir) to maintain virologic suppression while preserving TB efficacy [14,15,16,44]. Similar complexities arise with macrolides in MAC therapy, which can increase exposure to certain antiretrovirals [4,101]. These interactions demand tailored prescribing and vigilant monitoring of toxicities, including hepatotoxicity and neuropathy, which may be exacerbated when ART and antimycobacterial agents overlap [16,17].

IRIS represents another cross-cutting complication. In TB, paradoxical IRIS may cause fever, lymphadenitis, or worsening pulmonary infiltrates shortly after ART initiation. While usually self-limited, severe forms—particularly CNS TB-IRIS—can be fatal without corticosteroid therapy [38,42,102]. MAC-IRIS and leprosy-IRIS are also well documented, often presenting as unmasking of subclinical infection or acute neuritis [9,10,12,43]. These reactions highlight the tension between early ART (which improves survival) and the risk of excessive inflammation, underscoring the need for predictive biomarkers and preventive strategies [73,102].

Drug resistance adds further complexity. MDR- and XDR-TB require longer, more toxic regimens and have historically been associated with poor outcomes in PLWH [18,19,29]. However, therapeutic advances have changed this landscape. Bedaquiline (introduced 2012), delamanid (2015), and pretomanid (2019) are no longer experimental but are integrated into WHO-endorsed MDR-TB regimens, often combined with linezolid to create shorter, all-oral regimens [21,22,25,76,78,80]. Although effective, emerging resistance to these drugs is already reported [75,78]. By contrast, MAC treatment still relies on macrolide–ethambutol backbones, with clinical success linked primarily to immune recovery on ART [4,26,101]. Leprosy management remains unchanged, with WHO multidrug therapy effective in HIV co-infection, though IRIS-related reactions after ART must be anticipated [9,10,12,20].

Host-directed therapies (HDTs) are an important frontier. Vitamin D supplementation has shown variable benefit in enhancing macrophage killing of *M. tuberculosis* [45,52]. Metformin promotes autophagy and alters macrophage metabolism, with epidemiologic studies linking its use to improved TB outcomes [50,54,92]. mTOR inhibitors like rapamycin, although immunosuppressive, improve granuloma structure and pathogen control in murine models [58,59]. Cytokine-based interventions—including interferon-γ, GM-CSF, and IL-7—have demonstrated potential to augment immune clearance and restore T-cell function in co-infected patients [62,63,64,65,66,67,68,69,70,72]. Importantly, deregulated host transcription factors such as NFAT5, NF-κB, HIF-1α, and TFEB are emerging as promising targets. For example, pharmacologic activation of TFEB enhanced rifampin efficacy by promoting autophagy in macrophages [27,90,91]. These data support combining antimicrobials with HDTs to improve cure rates and reduce post-treatment sequelae [51,101].

Prevention and integration of care are equally critical. Scaling up TB preventive therapy (e.g., isoniazid or rifapentine regimens) in PLWH can reduce incidence, with shorter regimens improving feasibility [14]. Integration of HIV and TB services, as recommended by WHO, enhances screening, treatment initiation, and adherence [98]. Vaccine development adds another layer of promise: the M72/AS01E vaccine demonstrated ~50% efficacy in preventing progression from latent to active TB, and recombinant vaccines like MTBVAC and VPM1002 are advancing toward large-scale trials [81,82,83,84,101]. For leprosy, chemoprophylaxis with single-dose rifampicin in household contacts—including PLWH—remains an implementation priority [20].

In summary, TB continues to drive mortality in PLWH, but MAC and leprosy illustrate the broader spectrum of mycobacterial co-infections that require tailored strategies. Across all three, clinicians must balance ART optimization, antimicrobial resistance, and the risk of IRIS. Established MDR-TB drugs (bedaquiline, delamanid, pretomanid, fluoroquinolones) are improving outcomes, while host-directed and immunomodulatory therapies open new avenues for adjunctive care. Future progress hinges on integrated service delivery, personalized care models, and preventive innovations, including vaccines and HDTs, to close the survival gap for HIV–mycobacteria co-infected patients. Contribution and outlook. In contrast to guideline-oriented reviews [103,104], this article situates HIV-associated TB within the broader spectrum of HIV-associated mycobacterial disease by explicitly incorporating MAC and leprosy and their IRIS-linked management. We synthesize established and emerging approaches with emphasis on host–pathogen interactions and host-directed/adjunctive strategies (e.g., metformin, glutathione augmentation, mTOR modulation, cytokine therapies, and vaccine candidates), mapping their ART/DDI interfaces and practical fit for PLWH. Future priorities include (i) mechanistic trials of HDTs tailored to ART regimens and IRIS risk, (ii) regulatory pathways that accommodate adjuncts alongside standard antimicrobials, and (iii) implementation research to operationalize these strategies within integrated HIV–TB/NTM/leprosy services in high-burden settings.

## Figures and Tables

**Table 1:** HIV Stage vs. Mycobacterial Manifestations and Outcomes.

HIV Disease Stage	Tuberculosis (TB)	Disseminated MAC	Leprosy (*M. leprae*)
Early HIV (CD4 > ~350/μL)	TB often presents with “classic” pulmonary disease (upper-lobe cavities, cough, fevers, night sweats), resembling HIV-negative TB [1,2]. Treatment success is high for drug-susceptible TB with standard first-line therapy (isoniazid, a rifamycin, pyrazinamide, ethambutol), with regimen choice/duration guided by susceptibility testing and major guidelines [3]. Drug-resistant TB requires second-line, all-oral regimens per WHO guidance (add WHO DR-TB guideline separately if you want WHO cited explicitly). Outcomes are good when immune function is largely intact [2,3].	Rare at preserved CD4; pulmonary MAC typically occurs with underlying lung disease. Disseminated disease is uncommon when CD4 is relatively preserved [4,5].	Leprosy risk is not increased by early HIV. Clinical presentation and response to standard multidrug therapy are unchanged. Outcomes mirror those in HIV-negative patients, assuming no severe immunosuppression.
Advanced HIV (CD4 < 200/μL)	TB risk and severity rise as CD4 falls. High rates of extrapulmonary and disseminated TB occur when CD4 < 50/μL. TB may present atypically (e.g., diffuse infiltrates, extrapulmonary sites). Mortality is higher (up to ~3-fold during TB therapy) without ART. Outcomes are poor if untreated, but can improve substantially with timely TB treatment and ART.	Incidence of disseminated MAC escalates at low CD4 counts (<50/μL). Presents with non-specific systemic symptoms (fever, weight loss, diarrhea, anemia) and multi-organ involvement. Without ART, MAC requires chronic suppressive therapy and carries high relapse risk.	Co-infection remains uncommon even in advanced HIV. Severe immunosuppression may blunt granulomatous inflammation, potentially leading to anergic (diffuse, lepromatous) leprosy forms—though studies have not found markedly altered leprosy spectra. Standard leprosy therapy still achieves high cure rates. However, advanced HIV patients are vulnerable to neuropathy and other infections that can complicate leprosy management.
After ART Initiation (IRIS phase)	Paradoxical TB-IRIS can occur within weeks of ART start in ~10–20% of co-treated patients, especially those with high TB antigen burden. Patients may acutely worsen (fever, enlarging lymph nodes or lesions) despite effective TB therapy. TB-IRIS is usually self-limited but can be severe (e.g., CNS TB-IRIS). Corticosteroids are used for moderate/severe cases to dampen inflammation. Most patients eventually improve with continued TB treatment and ART.	Unmasking MAC-IRIS is less common but can occur if subclinical MAC was present prior to ART. It typically manifests as fever, lymphadenitis, or focal disease flares shortly after ART commencement. Management includes NSAIDs for mild symptoms or a short prednisone course for severe inflammation. Importantly, ART is continued alongside ongoing MAC therapy. Prognosis: MAC-IRIS is generally manageable and rarely life-threatening; controlling HIV with ART ultimately improves MAC outcomes.	IRIS-leprosy: Immune reconstitution can trigger leprosy reversal reactions (Type 1 inflammation) or erythema nodosum leprosum (Type 2) in patients on or completing leprosy therapy. New leprosy diagnoses have occurred post-ART (unmasking of previously silent infections). These reactions cause acute nerve inflammation, requiring prompt anti-inflammatory treatment (corticosteroids) to prevent permanent nerve damage. Despite IRIS, leprosy treatment should be continued. With appropriate management of reactions, outcomes remain favorable (HIV does not significantly worsen long-term leprosy prognosis).

HIV Disease Stage and Mycobacterial Manifestations. Summarizes characteristic presentations, prognoses, and immune interactions of tuberculosis (TB), disseminated *Mycobacterium avium* complex (MAC), and leprosy (*M. leprae*) across early HIV infection, advanced immunosuppression, and after antiretroviral therapy (ART) initiation. Clinical outcomes, IRIS risk, and diagnostic challenges are emphasized relative to CD4+ T cell counts. Sources: Adapted from WHO and CDC guidelines, NIH OI guidance, and cohort studies of TB, MAC, and leprosy in HIV [1,2,3,4,5,6,7].

**Table 2:** Comparison of TB, MAC, and Leprosy Before vs. After Antiretroviral Therapy (ART).

Infection	Without ART (Untreated or Late HIV)	With Effective ART (After Initiation)
Tuberculosis (TB)	High burden and high mortality: TB thrives as CD4 declines, often disseminating in late-stage HIV. Without ART, patients face a ~15–20× higher TB risk and up to three-fold higher mortality during TB treatment. Standard 6-month TB therapy may succeed initially, but without HIV control, recurrent TB or new infections are common. Drug interactions go unmanaged, as rifampicin lowers levels of protease inhibitors and some NNRTIs potentially compromising HIV therapy if continued. Adherence is challenging due to heavy pill burden and side effects on top of untreated HIV.	Improved outcomes, with IRIS risk: Initiating ART dramatically reduces TB mortality and future reactivation risk, and allows immune reconstitution to contain TB. However, early ART can precipitatea transient inflammatory worsening of TB symptoms (TB-IRS). Management of co-treatment is crucial: rifampicin-based TB therapy requires adjusting the ART regimen (e.g., using rifabutin or double-dose integrase inhibitors) to avoid drug–drug interactions. With ART, TB treatment success rates improve, relapse rates drop, and long-term survival is significantly higher than in patients who remain ART-naïve.
MAC	Disseminated disease almost inevitable in late AIDS: Without ART, patients with CD4 < 50/μL are highly susceptible to MAC; prophylactic azithromycin is indicated in this scenario. If MAC infection occurs, therapy (macrolide + ethambutol ± third agent) must be continued indefinitely or until immune recovery, because relapse is likely when immune defenses remain low. Prognosis is poor in the absence of ART asMAC was historically a major cause of wasting and death in advanced AIDS. Drug interactions are less problematic than TB (macrolides have moderate interactions), but untreated HIV typically precludes MAC cure, as the infection will recur once antibiotics stop.	Prevention and faster clearance: Effective ART raises CD4 counts, drastically cutting MAC incidence, such thatroutine primary prophylaxis is no longer needed if HIV is promptly controlled. In co-infections, starting ART enables eventual discontinuation of MAC therapy after ≥12 months, once CD4 > 100/μL is sustained. ART also improves weight gain and anemia associated with disseminated MAC. Immune reconstitution can cause mild MAC-IRIS (fever, lymph node inflammation), but these events are manageable and outweighed by the benefit of immune recovery. Overall, patients on ART have far better MAC outcomes: higher cure rates, lower risk of relapse, and improved survival, transforming MAC from a fatal illness into a treatable infection in the ART era.
Leprosy	Similar course, but diagnosis often missed: In ART-naïve HIV patients, leprosy follows its usual spectrumranging from paucibacillary to multibacillary disease with no clear increase in frequency. Advanced immunosuppression might allow higher bacillary loads (lepromatous leprosy), but paradoxically, anergic leprosy is not dramatically more common than in HIV-negative cases. The standard 6–12 month MDT (rifampicin, dapsone, clofazimine) is effective and well-tolerated even without ART, and HIV co-infection alone doesn’t justify extending therapy. However, clinicians may fail to diagnose leprosy in HIV patients, confusing neuropathic symptoms with HIV neuropathy or other dermatological conditions. Without ART, any concurrent infections or malnutrition can complicate leprosy management.	IRIS and enhanced inflammatory responses: ART does not impair leprosy drug efficacy coinfected patients respond as well as those without HIV. But immune reconstitution frequently triggers leprosy IRIS reactions. Patients on ART may develop sudden nerve pain, skin inflammation, or new lesions (often Type 1 reversal reactions) within months of starting therapy. Managing these episodes with corticosteroids is critical to prevent permanent nerve damage. The timing of leprosy diagnosis can also shift: previously unrecognized cases may “unmask” after ART initiates, due to the recovering immune system mounting a response. Despite these challenges, prognosis with ART remains positive leprosy treatment outcomes and relapse rates are comparable to HIV-negative cases. In fact, effective HIV control likely aids long-term leprosy immunity. The key is vigilant monitoring for IRIS and interdisciplinary care (dermatology/HIV) during the initial ART period.

Impact of Antiretroviral Therapy (ART) on Mycobacterial Infection Management. Contrasts pre-ART and post-ART clinical courses for TB, MAC, and leprosy. Includes risk of dissemination, immune reconstitution inflammatory syndrome (IRIS), drug–drug interactions, and long-term outcomes. ART substantially improves infection control and prognosis but may introduce inflammatory complications requiring co-management. Sources: Based on WHO recommendations, IAS–USA guidelines, and comparative analyses of TB, MAC, and leprosy treatment outcomes in PLWH [14,15,16,17,18,19,20,21,22,23,24,25,26,27].

**Table 3:** ART–Rifamycin Interaction Summary.

Drug Class	Key Drugs	Interaction Mechanism/Effect	Clinical Management	Metabolic Effect
Rifamycins	Rifampin, Rifabutin, Rifapentine	Rifampin: A potent inducer of CYP3A4, UGT1A1, and P-gp; **reduces** the plasma concentrations of many antiretrovirals. Rifabutin: Milder inducer; interaction 60–80% less than rifampin. Rifapentine: Similarly to rifampin; weekly or daily high-dose regimens markedly lower ART levels except EFV.	Prefer rifabutin (150 mg 3×/week with boosted PIs; 300 mg daily otherwise). Avoid rifampin/rifapentine when compatible ART switch is not feasible; or switch ART to EFV or double-dose DTG/RAL.	Inducer (CYP3A4, UGT1A1, P-gp)
Protease Inhibitors (PIs)	Lopinavir, Atazanavir, Darunavir, Ritonavir, Cobicistat	Rifampin: Decreases protease inhibitor (PI) exposure, with PI AUC reduced by approximately 80–95%. This reduction can lead to virologic failure. These CYP3A4 inhibitors substantially increase rifampin concentrations, increasing the risk of serious hepatotoxicity. Coadministration should be avoided.	Contraindicate rifampin with standard PI regimens. Substitute rifabutin and reduce rifabutin dose (150 mg 3×/week) when PIs must be retained. Consider switching to INSTI-based ART if rifampin cannot be avoided.	PIs: CYP3A4 inhibitors/substrates
NNRTIs	Efavirenz (EFV), Nevirapine (NVP), Rilpivirine (RPV), Etravirine (ETR), Doravirine (DOR), Delavirdine	EFV: Rifampin reduces EFV AUC by about 20 percent. The usual 600 mg once daily dose is generally adequate. An 800 mg dose is not used in routine practice. NVP: Rifampin markedly reduces NVP exposure and increases the risk of hepatotoxicity. Avoid coadministration. RPV, ETR, DOR: Rifampin lowers exposure by more than 80 percent. Coadministration is contraindicated. Delavirdine: Strong CYP3A inhibitor; unpredictable with rifamycins.	Maintain EFV 600 mg with rifampin (guideline-preferred co-therapy). Switch NVP to EFV or use rifabutin. Avoid RPV, ETR, DOR with rifampin; DOR 100 mg BID permitted with rifabutin. Never combine delavirdine with any rifamycin.	EFV: CYP3A inducer; Others: CYP3A substrates; Delavirdine: CYP3A inhibitor
INSTIs	Dolutegravir (DTG), Raltegravir (RAL), Bictegravir (BIC), Elvitegravir/cobicistat (EVG/c), Cabotegravir (CAB)	Rifampin induces UGT1A1 and CYP3A, which **reduces dolutegravir (DTG) exposure** (AUC decreases by ~54%). It also **reduces raltegravir (RAL) exposure**, with trough concentrations decreasing by ~40%. Rifampin essentially abolishes BIC & EVG/c exposures; CAB also at risk. Rifabutin has minimal effect on DTG & RAL.	Double DTG to 50 mg BID or RAL to 800 mg BID when co-administered with rifampin. Do NOT use BIC or EVG/c with rifampin; switch to DTG/RAL or replace rifampin with rifabutin. Avoid long-acting CAB/RPV with rifamycins; postpone injections until rifamycin therapy complete.	Rifampin: UGT1A1/CYP3A inducer; EVG/c: CYP3A inhibitor (via cobicistat)

Drug–drug interactions between rifamycin antibiotics and major antiretroviral therapy (ART) classes. This table summarizes interaction mechanisms, metabolic pathways (CYP3A4, UGT1A1, P-gp), and evidence-based management across antiretroviral therapy classes. Overall, rifabutin provides the most compatible coadministration profile, while rifampin substantially lowers plasma concentrations of most protease inhibitors, most non-nucleoside reverse transcriptase inhibitors except efavirenz, and several integrase strand transfer inhibitors [15,16,28]. With protease inhibitors, rifampin reduces exposure by approximately 80 to 95 percent, increasing the risk of virologic failure, and pharmacokinetic boosters such as ritonavir or cobicistat can markedly raise rifampin levels and hepatotoxicity risk; rifampin should therefore be avoided with protease inhibitor–based regimens, and if a protease inhibitor must be continued, rifabutin with dose reduction, for example 150 mg three times weekly with boosted protease inhibitors, is preferred [15,16]. Among NNRTIs, efavirenz with rifampin produces about a 20 percent reduction in efavirenz exposure but the 600 mg once-daily dose remains guideline-preferred; nevirapine should be avoided with rifampin because of reduced levels and increased hepatotoxicity; rifampin markedly lowers rilpivirine, etravirine, and doravirine exposure by more than 80 percent; doravirine 100 mg twice daily is acceptable with rifabutin; delavirdine should be avoided with any rifamycin [15,16]. For INSTIs, rifampin induction of UGT1A1 and CYP3A reduces dolutegravir exposure by approximately 50 to 60 percent and lowers raltegravir trough concentrations by about 40 percent; management includes dolutegravir 50 mg twice daily or raltegravir 800 mg twice daily when coadministered with rifampin; bictegravir and elvitegravir boosted with cobicistat should not be used with rifampin; rifabutin has minimal effect on dolutegravir and raltegravir; long-acting cabotegravir and rilpivirine injections should be avoided during rifamycin therapy and deferred until the rifamycin course is completed [15,16]. Programmatic timing decisions, such as when to switch to efavirenz, when to use twice-daily dolutegravir or high-dose raltegravir with rifampin, and when to prefer rifabutin over rifampin during concurrent TB and HIV treatment, should follow consolidated national and international guidance [12,14,16].

## Data Availability

No new data were created or analyzed in this study. Data sharing is not applicable to this article.
